# The Relationship Between Processed Food Consumption and Periodontal Disease: Sex Disparities in the Majorcan Adolescent Population

**DOI:** 10.3390/life15040580

**Published:** 2025-04-01

**Authors:** Irene Coll, Daniela Vallejos, Pablo Estebala, Nora López-Safont

**Affiliations:** 1 Faculty of Dentistry, University ADEMA School, C. Passamaners 11, 07009 Palma, Spain; i.coll@eua.edu.es (I.C.); d.vallejos@eua.edu.es (D.V.); p.estebala@eua.edu.es (P.E.); 2 Health Group of University Institute for Research in Health Sciences (IUNICS), Ctra. Valldemossa Km 7.5, 07122 Palma, Spain; 3 Biology Department, University of Balearics Islands, Ctra. Valldemossa Km 7.5, 07122 Palma, Spain

**Keywords:** periodontal disease, consumption frequency, processed food

## Abstract

Background: The diet of young people in Spain has changed significantly, with a departure from a balanced dietary pattern and a greater intake of processed foods. Such food generates an acidic environment in the mouth, which promotes the multiplication of bacteria capable of causing inflammation and damage to the gums. Aim: This study aimed to determine the association between the frequency of consuming processed foods and periodontal disease, as well as sex differences, in an adolescent population. Methods: A study was conducted on 233 students aged 15 to examine the frequency of food consumption and its correlation with periodontal disease. Differences were determined via a Student’s *t*-test to compare the means. A chi-square test was used to compare categorical variables. The 95% confidence interval estimate was used in all cases (*p* < 0.05). Results: It was observed that girls have a higher mean number of healthy sextants than boys (3.26 ± 0.20 vs. 2.70 ± 0.21; *p* = 0.029). A statistically significant difference was noted between healthy and affected subjects in the frequency of consumption of packaged milkshakes (*p* = 0.003), industrial juices (*p* = 0.009), industrial pastries (*p* = 0.018), and fruits in syrup (*p* = 0.022). When segmented by sex, a statistically significant difference was noted in boys between healthy and affected subjects in the frequency of consumption of packaged milkshakes (*p* = 0.044), salty snacks (*p* = 0.032), and cold cuts (*p* = 0.033); in girls, the difference was detected in industrial juices (0.024). Conclusions: The results of this study suggest that adolescent boys are more affected periodontally than girls. In both sexes, the level of consumption of processed foods affects the presence of periodontal disease.

## 1. Introduction

The World Health Organization (WHO) defines adolescence as the period of human growth and development that occurs after childhood and before adulthood, between the ages of 10 and 19 [1]. Adolescence is the period of accelerated biological growth, changes, and social role transitions that bridges the gap between childhood and adulthood [1]. The adolescent years are particularly critical for the establishment of lifestyles, during which the reinforcement of certain childhood habits occurs with the adoption of new ones learned through socialization scenarios [2,3].

Oral health is an integral part of healthy living and vital to quality of life, affecting physical and emotional well-being, appearance, and interpersonal relationships [4]. Oral diseases continue to impose a significant global burden [5]. Dental caries is the most prevalent oral and non-communicable disease (NCD) globally, with 2.3 billion people suffering from caries of permanent teeth and more than 530 million children suffering from caries of primary teeth [6]. According to the Global Burden of Disease Study (2016), severe periodontal disease was the 11th most prevalent condition in the world [7]. The prevalence of periodontal disease was reported to range from 20% to 50% around the world [8].

Growing evidence has shown that the frequent consumption of ultra-processed food (UPF) is extremely harmful to health [9], contributing to increasing the risk of several diet-related chronic diseases [10,11]. Nutrition and oral health are closely related [4]. Changing food patterns have been indicated to influence oral health development globally [4].

Adolescence may be a critical period for periodontal status [12]. Epidemiological and immunological data suggest that irreversible tissue damage from periodontal disease begins in late adolescence and early adulthood [13,14,15].

Gingivitis is becoming frequent in adolescents [16,17].

The latest data available in Spain regarding the prevalence of periodontal diseases date from the last survey conducted in 2020: in the 12- and 15-year-old groups, the percentage of individuals with calculus was 28% and 34%; in the 35–44-year-old group, the percentage of subjects with deep pockets (≥6 mm) was 7.6%, and 11.6% had periodontal pockets of 4–5 mm; in the 65–74-year-old group, these values were 17.9% and 22.9%, respectively. In terms of sex, a significant association was found at 12, 35–44, and 65–74 years of age, where the mean number of healthy sextants was higher in women than in men in all the groups [18].

On the Balearic Islands, the last oral health survey dates from 2005, in which the percentage of subjects with healthy sextants (without periodontal disease) was 57.6% at the age of 7 years, 52.2% at the age of 12 years, and 49.1% at the age of 14 years. The percentage of schoolchildren with bleeding sextants was 33.6% at the age of 7 years (33.6%), 28.8% at the age of 12 years, and 19.4% at the age of 14 years. The percentage of schoolchildren with sextants with calculus was 8.8% at the age of 7 years, 19% at the age of 12 years, and 31.5% at the age of 14 years. Sex only had an influence at the ages of 12 and 14 years, with a higher prevalence of schoolchildren with calculus in the male sex [19]. 

In a recent study in Mallorca, Balearic Islands, it was observed that students aged 15 years have a caries prevalence rate of 45.49%, higher than those aged 12 (27.39%). The presence of dental calculus in 15-year-old students is 52.8%, even higher than in 12-year-olds (30%).

Concerning the periodontal health of men and women, it has been noted that disparities in periodontal indices by sex have not been thoroughly examined, yet such disparities are likely associated with inadequate oral hygiene, a less favorable attitude towards oral health, and a reduced frequency of dental checkups among men [20,21].

In 2013, the European Food Safety Authority (EFSA) noted that 68% of young people consumed some type of energy drink. This study also indicated that 25% of young people had an excessive intake [22].

According to the NOVA classification system (which classifies foods according to the extent of their processing), Group 4 includes ultra-processed foods such as carbonated beverages, sugary drinks, bread rolls, biscuits, baked goods, cakes, pies, and many more foods whose matrix contains at least five ingredients or more [23].

This type of food produces an acidic environment in the mouth, which favors the multiplication of bacteria capable of causing inflammation and damage to the gingiva [16]. At the same time, unfavorable bacteria that can create an imbalance in the normal microbiota of the mouth continue to multiply [16]. Thus, gingival inflammation increases, and redness and bleeding appear in multiple areas of the gingiva; gingivitis is caused by dental plaque [24].

The causal relationship between sugars and dental caries is well established in the literature [25], but previous studies in the literature have also reported an association between added sugar intake [26], carbohydrate consumption [27], and periodontitis [28]. In a recent population-based study, it was also reported that an unhealthy diet with an increased consumption of fast food and manufactured products was associated with initial and moderate periodontitis in Brazilian adolescents [29].

Regarding sex and food consumption, the Health Behaviour in School-aged Children (HBSC) 2002–2006–2010 study shows that the daily consumption of sweets by girls is slightly higher than that of boys in the three editions, and the daily consumption of soft drinks or sugared beverages is slightly higher in boys than in girls [30].

Therefore, this study suggests that the high consumption of processed foods is associated with an increased risk of periodontal disease in adolescents and that there are gender differences in the magnitude of this relationship, being more pronounced in one of the sexes.

## 2. Materials and Methods

### 2.1. Study Design and Target Population

This cross-sectional observational epidemiological study was designed following the recommendations of the World Health Organization (WHO) for conducting oral health surveys using the Pathfinder methodology.

The school population was the target of the present study, with a group of an index age of 15 years, as recommended by the WHO. A total of 233 adolescents aged 15 years were analyzed (girls *n =* 121, boys *n* = 112). The information on the sampling sites, where the sample population was collected, was taken from the Directorate General for Planning, Organization and Centers of the Autonomous Community of the Balearic Islands and the National Institute of Statistics. The strata included were the population center (urban, peri-urban, and rural centers) and type of school (public and charter/private). After segmenting the population into various strata, systematic random sampling was used to select the schools, ensuring the representativeness of each stratum by applying the proportionality criterion according to the characteristics of the study area. This included the type of school, 190 students from public schools (81.5%) and 43 students from private/charter schools (18.4%), and the geographic location, 101 students from urban area schools (43.3%) and 132 students from rural area schools (56.6%). 

The criteria for the inclusion and exclusion of participants are described in Table 1.

The design, protocol, and methodology of this cross-sectional observational study are described in more detail elsewhere [31]. This study was carried out in adherence to the guidelines of the Strengthening the Reporting of Observational Studies (STROBE) statement. We have included this information in the Supplementary Files.

The present study was approved by The Research Ethics Committee of The Balearic Islands (CEI: IB3737/18) in accordance with the current legislation and was conducted in line with the principles contained in the Declaration of Helsinki and the standards of good clinical practice. Before starting this study, information was provided to the students’ parents or guardians (they received the study information sheet and the informed consent form), and only those students whose parents or guardians signed the consent form were included in this study.

### 2.2. Data Collection and Study Variables

The oral health (CPI, number of sextants in each code, periodontal status), sex, and frequency of consumption of processed foods variables were recorded between November 2018 and December 2019. The oral health data were extracted from “Oral Health Surveys: Basic Methods” [32] using standardized lighting conditions (headlight), instruments (dental mouth mirror #5 and WHO periodontal probe (Madrid, Spain)), and examinee positions. The data collected on the frequency of consumption were extracted based on the guidelines proposed by the European Food Safety Authority (EFSA). These guidelines are part of the “EUMenu Project” in Europe [33] and are included in the 2009 European methodological guide, “General Principles for the Collection of National Food Consumption Data in the View of a pan-European Dietary Survey” [34]. The survey design, protocol, and methodology have already been described elsewhere [35].

The foods and beverages analyzed were classified according to the four NOVA food categories:

- Group 1: Natural and minimally processed foods: Unprocessed or natural foods are obtained directly from plants or animals and do not undergo any alteration following their removal from nature. Minimally processed foods are natural foods that have been submitted to cleaning, the removal of inedible or unwanted parts, fractioning, grinding, drying, fermentation, pasteurization, cooling, freezing, or other processes that may subtract part of the food but which do not add oils, fats, sugar, salt, or other substances to the original food (eggs, nuts, peanuts, coffee).
- Group 2: Processed culinary ingredients: These are products extracted from natural foods or from nature by processes such as pressing, grinding, crushing, pulverizing, and refining. They are used in homes and restaurants to season and cook food and thus create varied and delicious dishes and meals of all types, including broths and soups, salads, pies, breads, cakes, sweets, and preserves (butter, lard, honey extracted from honeycombs).
- Group 3: Processed foods: These are products manufactured by industry with the use of salt, sugar, oil, or other substances (Group 2) added to natural or minimally processed foods (Group 1) to preserve or to make them more palatable. They are derived directly from foods and are recognized as versions of the original foods. They are usually consumed as a part of or as a side dish in culinary preparations made using natural or minimally processed foods. Most processed foods have two or three ingredients (bacon, tomato extract, pastes, or concentrates).
- Group 4: Ultra-processed foods and beverages: These are industrial formulations made entirely or mostly from substances extracted from foods (oils, fats, sugar, starch, and proteins), derived from food constituents (hydrogenated fats and modified starch), or synthesized in laboratories from food substrates or other organic sources (flavor enhancers, colors, and several food additives used to make the product hyper-palatable). Manufacturing techniques include extrusion, molding, and preprocessing by frying. Beverages may be ultra-processed. Group 1 foods are a small proportion of, or are even absent from, ultra-processed products (energy and sports drinks, biscuits, sweetened juices).

The variables considered for the analysis were sex, the CPI (community periodontal index), the number of sextants in each code (healthy, bleeding, and calculus), periodontal status (healthy or affected), and the frequency of consumption.

### 2.3. Statistical Analysis

The data were analyzed using the SPSS 27.0.1.0^®^ statistics applications. Numerical variables are expressed as the mean ± standard deviation, while nominal variables are expressed as percentages. Differences were determined via the Student’s *t*-test to compare the means. The chi-square test was used to compare categorical variables. To obtain a measure of precision (of the random error present in the data), the 95% confidence interval estimate (*p* < 0.05) was used in all cases.

## 3. Results

### 3.1. Sample Distribution

Data were collected between October 2018 and November 2019 from 233 subjects included in this study: 112 boys and 121 girls. The subjects were divided into two groups based on periodontal status: healthy (all sextants are healthy) or affected (one or more sextants are affected) (Table 2).

### 3.2. Level of Periodontal Disease in the Entire Sample

Table 3 shows the percentages of subjects in each maximum CPI (community periodontal index) code. Only the presence of calculus and bleeding on probing was assessed. The percentage of individuals with calculus or tartar was 52.8%; in contrast, healthy periodontium was found in 22.7%.

### 3.3. Number of Affected Sextants by Sex

Regarding the severity of the process, a significant difference was observed in the group of healthy sextants, with the mean in the group of girls being higher than in the group of boys (3.26 ± 2.21 vs. 2.70 ± 2.29; *p* = 0.029) (Table 4).

### 3.4. Consumption of Ultra-Processed Foods and the Presence of Periodontal Disease

A statistically significant difference was observed between healthy and affected subjects in the whole sample in relation to the frequency of consumption of packaged milkshakes (*p* = 0.003), industrial juices (*p* = 0.009), industrial pastries (*p* = 0.018), and fruit in syrup (*p* = 0.022) (Table 5).

A statistically significant difference was observed between healthy and affected boys in terms of the frequency of consumption of packaged milkshakes (*p* = 0.044), salty snacks (*p* = 0.032), fruit (*p* = 0.034), and cold cuts (*p* = 0.033) (Table 6).

A statistically significant difference was observed between healthy and affected girls in relation to the frequency of consumption of industrial juices (*p* = 0.024) (Table 6).

## 4. Discussion

Oral health is an integral part of general health and plays an important role in quality of life. During adolescence, behaviors that harm or promote health are modeled, so this stage of life is the best time to try to encourage responsibility for self-care and good habits [36].

Biological factors like age, sex, and hereditary factors are intrinsically unmodifiable. Social determinants are factors modifiable by behavioral or lifestyle changes [37]. Socioeconomic status is also directly related to oral hygiene practices. Adolescents living in resource-limited settings are more likely to be significantly affected by chronic oral diseases [38]. This can be explained by the fact that high-income families can successfully buffer adolescents from the adverse impacts of oral conditions [4].

Nutrition is a critical component of lifelong health and development [39]. Better nutrition improves the health of infants, children, and adults, reinforces the immune system, promotes safer pregnancy and childbirth, contributes to longevity, and reduces the risk of non-communicable diseases [40]. Today, the world is facing a double burden of malnutrition, including both undernutrition and overnutrition [41].

Increasing rates of overweight and obesity around the world are accompanied by soaring rates of chronic diseases (CDs) such as cardiovascular diseases (CVDs), diabetes, and cancer [42]. It is, however, important not to link malnutrition only with obesity and major CDs. Periodontal disease (PD) is a non-communicable disease with a 45–50% global prevalence, with 11% of the global population suffering from a severe form, which makes it the sixth most common disease [4,43].

Despite advances in the prevention and knowledge of the development of periodontal disease, it remains a significant public health issue, particularly among vulnerable populations like schoolchildren [44].

This study’s findings underscore the need for a proper diet for oral health, particularly in preventing periodontal disorders at early ages. This suggests the importance of starting good eating habits from childhood to avoid developing inflammatory diseases like periodontitis.

The foods that comprise a person’s diet are key to the onset of oral diseases [45,46,47]. Globally, an unhealthy diet, especially one high in free sugar, is a crucial risk factor for oral degradation [4]. The consumption of these free sugars—notably present in manufactured food—can be detrimental to oral health development as they cause dental decay in children and adolescents [4], but a variety of factors have also been linked with the etiology of periodontal disease [48]. The primary cause is poor oral hygiene, which leads to the formation of dental plaque containing microorganisms [49]. In addition to these local elements, a number of systemic factors such as diabetes, cardiovascular disease, pregnancy, and rheumatoid arthritis [50] have also been associated with periodontitis [49].

Studies indicate that dietary changes are crucial for improving both human health and environmental sustainability [12,14,15,51]. A high meat consumption and industrialized foods, prevalent in high-income countries, are linked to higher cancer rates and other non-communicable diseases (NCDs), such as diabetes and cardiovascular conditions [52].

The findings of this observational study conducted on the frequency of consumption of various specific food groups during school age in Mallorca align with the established correlation between the intake of ultra-processed foods and an increased prevalence of periodontal disease [41,45,53,54,55,56], indicating that children with a higher consumption of ultra-processed foods have poorer oral health. Recent studies suggest that men tend to consume more ultra-processed foods [57], more frequently and more regularly compared to women [58]. Women tend to be more concerned about health and weight control, which leads them to opt for foods perceived as healthier and avoid high-calorie or ultra-processed products [58].

In relation to the severity of the process, a significant difference has been noted according to sex in the group of healthy sextants, with the mean in the group of girls being higher than in the group of boys. Similar data were collected in the latest Oral Health Survey in Spain in 2020, where the mean of healthy sextants was also higher in girls (4.18 sextants) than in boys (3.62 sextants) [18].

Numerous studies indicate a higher prevalence of gingivitis in males [58,59,60], contradicting findings from research that suggest a predominance in females [61,62].

In our study, the intake of industrial pastries, packaged milkshakes, industrial juices, and fruit in syrup was associated with higher consumption among schoolchildren with periodontal disease, a finding also reported by other studies [28,41,62,63]. However, sugared beverages, isotonic drinks, and condiments were not significantly related to the development of periodontitis, although a positive trend was noted between their consumption and subjects with periodontitis.

Numerous studies indicate that the intake of ultra-processed foods correlates with a greater incidence of periodontal diseases in girls than in boys [64]; however, this finding is not universally supported across all studies [65], and certain studies did not find disparities in ultra-processed food consumption between the sexes [66]. In the segmentation by sex in our study, a statistically significant difference between healthy and affected subjects was observed in boys concerning the frequency of consumption of packaged milkshakes, salty snacks, fruit, and cold cuts; in girls, only the frequency of consumption of industrial juices was significant.

All foods are classified according to the NOVA system, which categorizes them into groups based on the extent and purpose of their processing. In this system, foods are classified as either “processed foods” or “ultra-processed foods” [23,67,68]. They all contain high amounts of sugar and saturated fats, as in the case of pastries, which are associated with an increased risk of developing diseases, including periodontitis or caries. The NOVA system provides a useful framework for understanding the role of food processing in dietary habits and its potential impact on oral health [28,69].

However, a systematic review by Sadler et al. [70] indicates insufficient evidence to assert that such foods increase the risk of oral diseases; thus, further research is needed to determine whether their consumption increases or decreases the risk of periodontitis or other oral conditions [70].

Ultra-processed foods, rich in sugars and saturated fats, low in fiber, and low in polyunsaturated fats, increase the risk of periodontal diseases [26,27,41]. Their inflammatory potential promotes chronic low-grade inflammation, which alters homeostasis [69] and increases the risk of developing inflammation-related diseases and pathologies such as periodontitis [28].

This study has some limitations. As cross-sectional study, it cannot establish causal relationships between processed food consumption and periodontal disease. A multivariate analysis could be performed. The reliance on self-reported data introduces potential recall bias or socially desirable responses. Furthermore, confounding factors such as oral hygiene, access to dental care, and socioeconomic status were not controlled for, which could influence the results. The diversity within processed food categories and variations in periodontal disease assessment methods could also affect the findings. Finally, hormonal changes during adolescence and individual genetic factors were not considered, which may impact the outcomes.

Despite these limitations, this study provides valuable insights into the relationship between dietary habits and periodontal health in adolescents. The sample size, while limited, allowed for a detailed analysis of food consumption patterns and periodontal status. The use of the NOVA classification system for food processing is a strength, as it offers a clear framework for assessing food quality and its potential impact on oral health. Additionally, the inclusion of both boys and girls allowed for an analysis of sex differences, contributing to a more comprehensive understanding of how gender may influence dietary habits and periodontal health.

In future studies, it would be valuable to expand the sample to include a more diverse population, better representing different socioeconomic and demographic groups. Additionally, factors affecting adolescent lifestyles, such as screen time, tobacco and alcohol use, stress related to studies, and oral hygiene habits, could be incorporated. These factors may influence diet and periodontal health, and analyzing how they interact would provide a more comprehensive understanding of the determinants of periodontal disease.

## 5. Conclusions

This study suggests an association between dietary habits and oral health in 15-year-old students, with boys showing a lower mean number of healthy sextants and higher processed food consumption compared to girls. The processed food intake is higher in students with affected sextants.

Our findings reinforce the need for public health efforts, interventions, and policies to reduce UPF consumption in order to improve the oral health of children and adolescents. This pattern suggests that educational interventions should consider gender differences to be more effective in promoting healthy eating habits.

## Figures and Tables

**Table 1:** Inclusion and exclusion criteria of participants.

Inclusion Criteria	Exclusion Criteria
Age	Age outside the range
School attendance	Severe systemic diseases
Informed consent	Unavailability or lack of cooperation

**Table 2:** Sample distribution.

		*n*	%
Sex	Boys	112	48.06
	Girls	121	51.93
Disease	Healthy	53	22.75
	Affected	177	75.96

(*n:* sample size).

**Table 3:** Maximum community periodontal index.

Maximum CPI	*n*	%	Mean	SD	SE	95% CI
15 years	194					
0 (healthy)	53	22.7%	2.97	2.26	0.14	(2.68–3.27)
1 (bleeding)	54	23.2%	1.97	1.92	0.12	(1.72–2.22)
2 (calculus)	87	52.80%	0.66	0.99	0.03	(0.54–0.80)
Not collected	3	-	0.04	0.047	0.06	(−0.1–0.10)
The 6 sextants are X	0	-				

(*n*: sample size; SD: standard deviation; SE: standard error; CI: confidence interval; X means excluded).

**Table 4:** Maximum community periodontal index (CPI). Mean number of sextants in each code.

CPI Code	Sex	*n*	Mean	SD	SE	95% CI	*p* Value
0 (healthy)	Boys	110	2.7	2.29	0.21	(2.26–3.13)	<0.029 *
	Girls	120	3.26	2.21	0.2	(2.86–3.66)	
≥1 (bleeding)	Boys	110	2.12	1.92	0.45	(1.76–2.49)	0.161
	Girls	120	1.87	1.93	0.17	(1.52–2.22)	
≥2 (calculus)	Boys	110	0.71	1.05	0.71	(0.51–0.91)	0.261
	Girls	120	0.63	0.95	0.63	(0.46–0.08)	
(X (excluded)	Boys	110	0.01	0.19	0.01	(0.00–0.05)	
	Girls	120	0	0	0.03	-	
Not collected	Boys	2	0.04	0.15	0.03	(0.00–0.11)	
	Girls	1	0	0		-	
The 6 sextants are X	Boys	0	-				-
	Girls	0	-				-

(*n*: sample size; SD: standard deviation; SE: standard error; CI: confidence interval; Student’s *t*-test: * variable with significant effect (*p* < 0.05); X means excluded).

**Table 5:** Frequency of consumption of processed foods that influence periodontal disease in the sample studied.

		*n*	%	Healthy	Affected	*p* Value
Packaged milkshakes (NOVA classification no. 4)	Monthly	164	72.2%	48	116	0.003 *
	Several times a week	33	14.5%	3	30	
	Once or several times a day	30	13.2%	2	28	
Industrial juices (NOVA classification no. 4)	Monthly	142	63.9%	41	101	0.009 *
	Several times a week	37	16.6%	7	30	
	Once or several times a day	43	19.3%	3	40	
Industrial pastries (NOVA classification no. 4)	Monthly	134	58.7%	40	94	0.018 *
	Several times a week	62	27.1%	9	53	
	Once or several times a day	32	14.0%	4	28	
Fruit in syrup (NOVA classification no. 3–4)	Monthly	184	81.7%	50	134	0.022 *
	Several times a week	18	8.0%	2	16	
	Once or several times a day	23	10.2%	1	22	
Sugared beverages (NOVA classification no. 4)	Monthly	142	62.8%	37	105	0.209
	Several times a week	50	22.1%	12	38	
	Once or several times a day	34	15.0%	4	30	
Isotonic drinks (NOVA classification no. 4)	Monthly	162	72.0%	44	118	0.069
	Several times a week	38	16.8%	5	33	
	Once or several times a day	25	11.1%	3	22	
Condiments (NOVA classification no. 4)	Monthly	125	55.5%	36	89	0.074
	Several times a week	66	29.3%	10	56	
	Once or several times a day	34	15.1%	6	28	
Salty snacks (NOVA classification no. 4)	Monthly	163	71.4%	44	119	0.104
	Several times a week	45	19.7%	6	29	
	Once or several times a day	20	8.7%	3	17	
Cold cuts (NOVA classification no. 3–4)	Monthly	115	50.4%	31	84	0.392
	Several times a week	75	32.8%	14	61	
	Once or several times a day	38	16.6%	8	30	

(*n*: mean, healthy, affected; chi-square: * variable with significant effect (*p* < 0.05)).

**Table 6:** Frequency of consumption of processed foods that influence periodontal disease by sex.

Processed Food	Boys							Girls						
	Monthly *n* (%)		Weekly *n* (%)		Daily *n* (%)		*p* Value	Monthly *n* (%)		Weekly *n* (%)		Daily *n* (%)		*p* Value
	Healthy	Affected	Healthy	Affected	Healthy	Affected		Healthy	Affected	Healthy	Affected	Healthy	Affected	
Packaged milkshakes (NOVA no. 4)	22 (28.5)	55 (71.4)	1 (5.8)	16 (94.1)	1 (7.1)	13 (92.8)	0.044 *	26 (29.8)	61 (70.1)	2 (12.5)	14 (87.5)	1 (6.2)	15 (93.7)	0.064
Industrial juices (NOVA no. 4)	17 (25.7)	49 (72.2)	4 (21.0)	15 (78.9)	2 (9.5)	19 (9.0)	0.290	24 (31.5)	52 (68.4)	3 (16.6)	15 (83.3)	1 (4.5)	21 (95.4)	0.024 *
Industrial pastries (NOVA no. 4)	19 (29.6)	45(70.3)	3 (11.1)	24 (88.8)	2 (11.1)	16 (88.8)	0.070	21 (30.0)	49 (70.0)	6 (17.1)	29 (82.8)	2 (14.2)	12 (85.7)	0.227
Fruit in syrup (NOVA no. 3–4)	22 (25.0)	66 (75.0)	1 (10.0)	9 (90.0)	2 (10.5)	17 (89.4)	0.306	28 (29.1)	68 (70.8)	1 (12.5)	7 (87.5)	0	12 (100.0)	0.062
Sugared beverages (NOVA no. 4)	14 (24.5)	43 (75.4)	8 (25.8)	23 (74.1)	2 (10.5)	17 (89.4)	0.387	23 (27.0)	62 (72.9)	4 (21.0)	15 (78.9)	2 (13.3)	13 (186.6)	0.487
Isotonic drinks (NOVA no. 4)	19 (26.3)	53 (73.6)	3 (11.5)	23 (88.4)	2 (22.2)	7 (77.7)	0.298	25 (27.7)	65 (72.2)	2 (16.6)	10 (83.3)	1 (6.2)	15 (93.7)	0.146
Condiments (NOVA no. 4)	18 (27.6)	47 (72.3)	3 (12.0)	22 (88.0)	3 (16.6)	15 (83.3)	0.074	18 (30.0)	42 (70.0)	7 (17.0)	34 (82.9)	3 (18.7)	13 (81.2)	0.285
Salty snacks (NOVA no. 4)	21 (27.6)	55 (72.3)	1 (3.7)	26 (96.2)	2 (28.5)	5 (71.4)	0.032 *	23 (26.4)	64 (73.5)	5 (27.7)	13 (72.2)	1 (7.6)	12 (92.3)	0.323
Cold cuts (NOVA no. 3–4	16 (33.3)	32 (66.6)	5 (11.1)	40 (88.8)	3 (18.7)	13 (81.2)	0.033 *	15 (28.8)	52 (70.0)	9 (30.0)	21 (70.0)	5 (22.7)	17 (77.7)	0.708

(*n*: mean, healthy, affected; chi-square: * variable with significant effect (*p* < 0.05)).

## Data Availability

The datasets generated and/or analyzed during the current study are not publicity available since we are working on them in other aspects but are available from the corresponding author upon reasonable request.

## Supplementary Materials

The following supporting information can be downloaded at: https://www.mdpi.com/article/10.3390/life15040580/s1, Table S1: STROBE Statement.
