# Supplementary material for: The Relationship Between Processed Food Consumption and Periodontal Disease: Sex Disparities in the Majorcan Adolescent Population
Source: Life (Basel). 2025 Apr 1;15(4):580. doi: 10.3390/life15040580 (PMC12028996; doi:10.3390/life15040580)
Supplement: Supplementary file 1 [file life-15-00580-s001.zip › life-3498387-supplementary.pdf]

Supplementary File Table S1: STROBE Statement

	Item No	Recommendation
**Title and abstract**	1	(*a*) Indicate the study's design with a commonly used term in the title or the abstract
The relationship between processed food consumption and periodontal disease: sex disparities in the Majorcan adolescent population.<br><br>**Abstract:** Background: The diet of young people in Spain has changed significantly, with a departure from a balanced dietary pattern and a greater intake of processed foods. Such food generates an acidic environment in the mouth, which promotes the multiplication of bacteria capable of causing inflammation and damage to the gums. Aim: This study aimed to determine the association between the frequency of consuming processed foods and periodontal disease, as well as sex differences, in an adolescent population. Methods: A study was conducted on 233 students aged 15 to examine the frequency of food consumption and its correlation with periodontal disease. Differences were determined via a Student's t-test to compare the means. A chi-square test was used to compare categorical variables. The 95% confidence interval estimate was used in all cases (p < 0.05). Results: It was observed that girls have a higher mean number of healthy sextants than boys (3.26± 0.20 vs. 2.70±0.21; P= 0.029). A statistically significant difference was noted between healthy and affected subjects in the frequency of consumption of packaged milkshakes (p=0.003), industrial juices (p=0.009), industrial pastries (p=0.018), and fruits in syrup (p=0.022). When segmented by sex, a statistically significant difference was noted in boys between healthy and affected subjects in the frequency of consumption of packaged milkshakes (p=0.044), salty snacks (p=0.032), and cold cuts (p=0.033); in girls, the difference was detected in industrial juices (0.02). Conclusions: The results of this study suggest that adolescent boys are more affected periodontally than girls. In both sexes, the level of consumption of processed foods affects the presence of periodontal disease.		(*b*) Provide in the abstract an informative and balanced summary of what was done and what was found
**Introduction**		
Adolescence (10-19 years) is a key stage of growth and habit formation, including those related to oral health [1]. Oral health is essential for well-being and quality of life, yet oral diseases remain a global issue [5,6]. Dental caries is the most prevalent oral disease, affecting 2.3 billion people [7], while severe periodontal disease is the eleventh most common condition worldwide [8], with a prevalence of 20-50% [9].<br>The frequent consumption of ultra-processed foods (UPF) is linked to chronic diseases and negatively impacts oral health [10-12]. These foods create an acidic	2	Explain the scientific background and rationale for the investigation being reported

environment in the mouth, promoting the growth of inflammatory bacteria and the development of gingivitis [18,26]. Adolescence may be a critical period for periodontal health, with evidence of irreversible tissue damage starting at this stage [2,15,16]. Gingivitis is increasingly common among adolescents [18,19].
In Spain, the National Oral Health Survey (2020) reported that 28-34% of adolescents aged 12 and 15 had dental calculus, while up to 22.9% of older adults had deep periodontal pockets [20]. In the Balearic Islands, 2005 data showed that at age 14, only 49.1% had healthy sextants, while 31.5% presented calculus [21]. A study in Mallorca found a caries prevalence of 45.49% among 15-year-olds, with 52.8% presenting dental calculus [21].
Gender differences in periodontal health remain underexplored, although men tend to have poorer oral hygiene habits and fewer dental visits [22,23]. Additionally, 68% of young people consume energy drinks, whose high sugar and acidity levels negatively impact oral health [24,25]. Previous studies have demonstrated a link between sugar-rich diets and periodontal diseases [27-31]. According to the HBSC study, girls consume more sweets, whereas boys consume more sugary soft drinks [32].

Objectives:<br><br>This study aimed to determine the association between the frequency of consuming processed foods and periodontal disease, as well as sex differences, in an adolescent population.<br><br>Therefore, this study suggests that the high consumption of processed foods is associated with an increased risk of periodontal disease in adolescents and that there are gender differences in the magnitude of this relationship, being more pronounced in one of the sexes.	3	State specific objectives, including any prespecified hypotheses
**Methods**		
Study design:<br>This cross-sectional observational epidemiological study was designed following the recommendations of the World Health Organization (WHO) for conducting oral health surveys using the Pathfinder methodology.<br>The present study was approved by The Research Ethics Committee of The Balearic Islands (CEI: IB3737/18).	4	Present key elements of study design early in the paper

Setting:<br>This study was conducted between October 2018 and December 2019. The strata included were the population center (urban, peri-urban, and rural centers) and type of school (public and charter/private). After segmenting the population into various strata, systematic random sampling was used to select the schools, ensuring the representativeness of each stratum by applying the proportionality criterion according to the characteristics of the study area.	5	Describe the setting, locations, and relevant dates, including periods of recruitment, exposure, follow-up, and data collection
Participants:<br>The school population was the target of the present study, with one group of an index age of 15 years, as recommended by the WHO. A total of 233 adolescents aged 15 years were analyzed (girls *n*= 121, boys *n* = 112): 190 students from public schools (81.5%) and 43 students from private/charter schools (18.4%). Regarding the geographic location, 101 students were from urban area schools (43.3%), and 132 students were from rural area schools (56.6%).<br>The criteria for the inclusion and exclusion of participants were as follows: participants must be within the specified age range, they must be attending school, informed consent must be obtained, they must be available to participate in this study, and they must have a general good health status. The exclusion criteria were as follows: individuals whose age falls outside the specified range, those with severe systemic diseases, and participants who are unavailable or lack cooperation.	6	(*a*) *Cohort study*—Give the eligibility criteria, and the sources and methods of selection of participants. Describe methods of follow-up<br>*Case–control study*—Give the eligibility criteria, and the sources and methods of case ascertainment and control selection. Give the rationale for the choice of cases and controls<br>*Cross-sectional study*—Give the eligibility criteria, and the sources and methods of selection of participants<br>(*b*) *Cohort study*—For matched studies, give matching criteria and number of exposed and unexposed<br>*Case–control study*—For matched studies, give matching criteria and the number of controls per case
Variables:<br><br>**Oral health:** assessed using the community periodontal index (CPI), recording the number of sextants in each CPI code to determine periodontal status.<br>**Sex:** categorized as male or female. **Frequency of processed food consumption:** evaluated based on self-reported dietary habits, categorizing consumption levels into specific frequency ranges.<br><br>**Outcomes: periodontal status:** determined using the CPI, which classifies periodontal health based on criteria such as bleeding on probing, the presence of calculus, shallow pockets (4-5 mm), and deep pockets (≥6 mm).<br><br>**Exposures: processed food consumption:** defined according to the frequency of intake of ultra-processed foods, including sugary beverages, sweets, and snacks.	7	Clearly define all outcomes, exposures, predictors, potential confounders, and effect modifiers. Give diagnostic criteria, if applicable

Predictors: dietary habits and oral hygiene practices, which may influence periodontal status.
Potential confounders: age, sex, socioeconomic status, and oral hygiene habits, as these factors can impact both dietary behaviors and oral health.
Effect modifiers: sex and age, which may influence the relationship between processed food consumption and periodontal health.
Diagnostic criteria: CPI scoring system was used for periodontal assessment, categorizing individuals based on the highest score recorded in any sextant: Code 0: healthy periodontium; Code 1: bleeding on probing; and Code 2: presence of calculus.

Data sources/ measurement: | 8* | For each variable of interest, give sources of data and details of methods of assessment (measurement). Describe comparability of assessment methods if there is more than one group

The variables of interest, including **oral health (CPI, number of sextants in each code, periodontal status), sex, and frequency of processed food consumption**, were recorded between **November 2018 and December 2019**.
Oral health assessment: Oral health data were collected following the **"Oral Health Surveys: Basic Methods"**, ensuring standardized examination conditions. The assessment was conducted using the following:

Standardized lighting conditions (headlight). Instruments**:** dental mouth mirror #5 and WHO periodontal probe.

Examinee positions: following WHO guidelines.

The c**ommunity periodontal index (CPI)** was used to assess periodontal status, recording the number of sextants in each code to determine the highest level of periodontal disease present.

Frequency of processed food consumption:

Data on food consumption frequency were extracted based on the **European Food Safety Authority (EFSA) guidelines**, as part of the **"EU Menu Project"** in Europe. These guidelines align with the **2009 European methodological guide**, "General Principles for the Collection of National Food Consumption Data in the View of a Pan-European Dietary Survey".

Classification of foods and beverages:

Foods and beverages were categorized according to the **NOVA food classification system**, which groups foods based on the extent of their processing:

- **Group 1: natural and minimally processed foods.**

- Directly obtained from plants or animals with minimal alterations (e.g., eggs, nuts, coffee).

- **Group 2: processed culinary ingredients.**
  - Extracted from natural foods, used for cooking and seasoning (e.g., butter, honey, lard).
- **Group 3: processed foods.**
  - Manufactured with added ingredients such as salt, sugar, or oil, often as preserved versions of natural foods (e.g., bacon, tomato extract).
- **Group 4: ultra-processed foods and beverages.**
  - Industrial formulations with multiple additives, food extracts, or synthesized ingredients (e.g., energy drinks, biscuits, sweetened juices).

This methodology ensured **comparability of assessment** by following internationally recognized procedures for oral health evaluation and dietary intake classification.

Bias:<br>To ensure the reliability and validity of this study, several measures were implemented to **minimize bias** in data collection, assessment, and analysis:<br>*1. Standardization of data collection.*<br><br>**Oral health assessment** followed the WHO **"Oral Health Surveys: Basic Methods"**, using **standardized lighting, instruments, and examinee positioning** to reduce **measurement bias**. **Periodontal examinations** were conducted with a **WHO periodontal probe** and a **dental mouth mirror #5**, ensuring uniform assessment criteria across all participants.<br><br>**Food consumption data** were collected using **guidelines from the European Food Safety Authority (EFSA)** and the **EU Menu Project**, aligning with the **2009 European methodological guide** to reduce variability in dietary reporting.	9	Describe any efforts to address potential sources of bias

2. Training and calibration of examiners: ***All examiners underwent training and calibration sessions before data collection to ensure inter- and intra-examiner reliability, reducing observer bias. A subset of participants was reassessed at different time points to verify the consistency of oral health evaluations.***

3. Use of objective diagnostic criteria: The community periodontal index (CPI) was employed to classify periodontal status based on standardized WHO criteria, reducing classification bias. Food categories were determined using the NOVA classification system, ensuring a consistent and recognized framework for dietary assessment.

4. Addressing recall bias: food consumption data were collected using structured food frequency questionnaires (FFQs) to reduce reliance on memory.

5. Minimizing selection bias: the random selection of schools was performed to ensure representative sampling of the adolescent population. Inclusion and exclusion criteria were clearly defined to avoid selective enrolment that could skew results.

These measures were implemented to **enhance the validity, reproducibility, and generalizability** of the study findings while minimizing **systematic errors and biases**.

Study size:	10	Explain how the study size was arrived at

The study size was determined based on statistical power calculations to ensure adequate representation of the target population and reliable detection of associations between oral health status, sex, and processed food consumption.
The following steps were carried out:
1. Sample size calculation.

The calculation was based on previous epidemiological data on periodontal health and dietary habits in adolescents.
The expected prevalence of periodontal disease and processed food consumption was obtained from prior studies and national surveys.
A confidence level of 95% and a margin of error of 5% were used to estimate the minimum required sample size.
A power of 80% was set to detect significant differences in periodontal health outcomes between exposure groups (e.g., high vs. low processed food consumption).
2. Adjustments for non-response and dropout.

To account for potential non-participation, missing data, and attrition, the sample size was increased by 10-15% beyond the minimum required number.
Strategies such as reminders and follow-ups were implemented to maximize participation and reduce dropout rates.

Quantitative variables:<br>In the study outlined, several variables were considered, including **oral health** (measured by CPI, sextants, and periodontal status), **sex**, and **frequency of consumption of processed foods**. These variables were analyzed using appropriate statistical methods to determine associations between diet and periodontal health in adolescents.<br><br>The following variables were chosen:<br><br>1. **Quantitative variables.**<br><br>**Quantitative variables** are those that are measured on a continuous scale or have numeric values. In this study, the following quantitative variables were considered:<br><br>• **CPI (community periodontal index)**: This is a measure of periodontal health, typically recorded as a score that reflects the presence of gum disease. The CPI helps assess the extent of periodontal disease and was treated as a continuous numerical variable.<br>• **Number of sextants in each code (healthy, bleeding, calculus)**: This refers to the number of sextants (sections of the mouth) that fall into different categories (healthy, bleeding, or with calculus). Each sextant was assigned a code representing the condition of the gums. This was treated as a numerical variable because it quantified the number of sextants affected by periodontal issues.<br>• **Frequency of consumption of processed foods**: This quantitative variable was based on the **guidelines of the European Food Safety Authority (EFSA)** and the **NOVA classification system**, which categorized foods into four groups (natural and minimally processed foods, processed culinary ingredients, processed foods, and ultra-processed foods). The frequency of consumption was measured on a scale (e.g., daily, weekly, or monthly consumption), which is a categorical approach for recording a typically continuous behavior. For statistical	11	Explain how quantitative variables were handled in the analyses. If applicable, describe which groupings were chosen and why

analysis, these frequencies were treated as quantitative data.

- **Statistical handling**:
  - **Numerical variables** (like CPI scores and the number of healthy/affected sextants) were expressed as **means ± standard deviations** to summarize the data, allowing for easy comparison across groups.
  - **Statistical significance** for these variables was assessed using the **Student's t-test** to compare the means between different groups (e.g., healthy vs. affected subjects, or boys vs. girls). The t-test is appropriate when comparing the means of two independent groups.
  - **Confidence intervals (CIs)**: a **95% confidence interval** was used in all cases to quantify the precision of the estimates and account for random error in the data.

2. **Categorical variables:**

Categorical variables are those that divide data into distinct categories or groups. In this study, several categorical variables were used:

- **Sex**: This was a categorical variable with two groups—**boys** and **girls**. It was important to analyze whether there were any sex differences in the relationship between processed food consumption and periodontal health.
- **Periodontal status (healthy vs. affected)**: This categorical variable classifies subjects based on the presence or absence of periodontal disease. Healthy participants are those who have no signs of gum disease, while affected participants exhibit symptoms of periodontal disease.
- **Frequency of consumption of processed foods**: As mentioned earlier, this variable was classified using the **NOVA system** (from natural foods to ultra-processed foods). The frequency was categorized and recorded as a nominal variable (e.g., never, sometimes, often, daily). These categories allowed for comparisons between groups with different consumption patterns.
- **Statistical handling**:
  - The **chi-square test** was used to assess the relationship between categorical

variables. This test is appropriate when comparing the frequencies or proportions of categories between two or more groups. For example, the chi-square test could compare how often processed foods are consumed by healthy vs. affected subjects or by boys vs. girls.

- **Nominal variables** like sex and periodontal status were presented as **percentages**, which is typical for categorical data. This allows for a clear comparison between different groups in terms of the proportion of participants within each category.

3. **Groupings chosen and their justification:**

- **Sex-based grouping**: This study divided the participants by sex (boys vs. girls) to examine whether the association between processed food consumption and periodontal health varied between the genders. This grouping is crucial because previous research suggests that boys and girls might have different dietary habits and health outcomes.
- **Periodontal health (healthy vs. affected)**: By dividing participants into healthy and affected groups, this study aimed to analyze the relationship between the frequency of processed food consumption and periodontal disease. This allowed for direct comparisons of food consumption habits between those with healthy gums and those with gum disease.
- **Frequency of processed food consumption**: The food consumption data were grouped according to the **NOVA food categories**. Grouping foods into these categories (e.g., ultra-processed vs. minimally processed) aligns with contemporary research on how different types of food impact health outcomes like periodontal disease. The NOVA classification helps capture the degree of food processing, which is important for understanding how different food types contribute to health conditions.

Statistical methods:	12	(*a*) Describe all statistical methods, including those used to control for confounding

This study used **SPSS software (version 27.0.1.0)** to conduct the statistical analysis. Numerical variables were presented with descriptive statistics (mean ± standard deviation), while categorical variables were presented as percentages.

The **Student's t-test** was used to compare means for continuous variables (e.g., CPI, healthy sextants) between two groups, such as healthy vs. affected individuals or boys vs. girls.

The **chi-square test** was employed to compare the frequencies or proportions of categorical variables (e.g., the consumption of different food types) between groups.

The **95% confidence interval** was used to express the precision of the results, ensuring that the estimates made by the statistical tests are reliable and account for random variability.

(*b*) Describe any methods used to examine subgroups and interactions

(*c*) Explain how missing data were addressed

(*d*) *Cohort study*—If applicable, explain how loss to follow-up was addressed
Case–control study—If applicable, explain how matching of cases and controls was addressed
Cross-sectional study—If applicable, describe analytical methods taking account of sampling strategy

(*e*) Describe any sensitivity analyses

Continued on next page

Results

Participants:

a) This study analyzed a group of **233 adolescents** aged **15 years**, with **girls (n=121)** and **boys (n=112)**. The following details are presented based on the study design:

- **Total potentially eligible individuals**: 233 adolescents aged 15 years.
- **Number examined for eligibility**: all 233 students were examined for eligibility based on the inclusion and exclusion criteria.
- **Number confirmed eligible**: after screening, all 233 adolescents were confirmed as eligible for this study, as they met the inclusion criteria (e.g., age, school attendance, informed consent, and availability to participate).
- **Number included in this study**: The 233 adolescents who were confirmed as eligible were included in this study. They represented a mix of both **public** (81.5%) and **private/charter** (18.4%) school students and came from **urban** (43.3%) and **rural** (56.6%) areas.
- **Number completing follow-up**: This study is cross-sectional, so there was no follow-up stage. All 233 participants were evaluated once during this study.
- **Number analyzed**: data from all 233 participants were analyzed, as no significant exclusion occurred during this study.

b) While **233 adolescents** were initially included based on the inclusion and exclusion criteria, potential reasons for non-participation could be as follows:

- **Exclusion during eligibility**:
  - Adolescents who were **outside the age range** of 15 years.
  - Those who had **severe systemic diseases**, as they would not meet the health criteria for participation.
  - Students who **did not provide informed consent** or were **unavailable** or **uncooperative** during the recruitment process.

These reasons for exclusion may have led to some adolescents not participating in this study, though the exact number of non-participants at each stage was not specified.

┌──────────────────────────┐
| 233 Potential Participants (aged 15) |
└──────────────────────────┘

↓

┌──────────────────────────┐
| 233 Examined for Eligibility |
└──────────────────────────┘

↓

13*

(a) Report numbers of individuals at each stage of study—eg numbers potentially eligible, examined for eligibility, confirmed eligible, included in the study, completing follow-up, and analysed

(b) Give reasons for non-participation at each stage

(c) Consider use of a flow diagram

233 Confirmed Eligible

↓

233 Included in this Study

↓

233 Analyzed (Cross-Sectional Study)

Descriptive data:

This study included **233 adolescents aged 15 years** (girls n=121, boys n=112, with the following characteristics:

Demographic information:

- **Age**: all participants were aged 15 years, as per the inclusion criteria.
- **Sex**: this study included a total of 121 **girls** (51.9%) and 112 **boys** (48.0%).

School type:

- **Public schools**: 190 students (81.5% of the total sample).
- **Private/charter schools**: 43 students (18.4% of the total sample).

Geographic location:

- **Urban areas**: 101 students (43.3% of the total sample).
- **Rural areas**: 132 students (56.6% of the total sample).

Clinical characteristics:

- **Oral health**: clinical data related to oral health were collected using the community periodontal index (CPI), the number of sextants in each code (healthy, bleeding, and calculus), and periodontal status (healthy or affected).
- **Periodontal status**: adolescents' periodontal health was assessed with a focus on those classified as "healthy" or "affected."

14*

(a) Give characteristics of study participants (eg demographic, clinical, social) and information on exposures and potential confounders

(b) Indicate number of participants with missing data for each variable of interest

(c) *Cohort study*—Summarise follow-up time (eg, average and total amount)

Outcome data:<br><br>Since this is a **cross-sectional study**, the data were collected at a single point in time, with no follow-up involved. The primary and secondary outcomes were measured simultaneously, and the outcomes represent the **status of periodontal health** and the **frequency of processed food consumption** at the time of assessment.	15*	*Cohort study*—Report numbers of outcome events or summary measures over time<br>*Case–control study*—Report numbers in each exposure category, or summary measures of exposure<br>*Cross-sectional study*—Report numbers of outcome events or summary measures
Main results:<br><br>***1. Unadjusted estimates:***<br><br>• **Periodontal health (primary outcome)**:<br>o The **unadjusted estimate** for the mean number of **healthy sextants** was calculated by comparing the **periodontal status** between adolescents with healthy gums versus those affected by periodontal disease.<br>o **Girls**:<br>▪ Mean number of healthy sextants = **3.26 ± 0.20**.<br>o **Boys**:<br>▪ Mean number of healthy sextants = **2.70 ± 0.20**.<br>o A **significant difference** was observed between boys and girls (p = 0.029), with girls having a higher mean number of healthy sextants.<br>• **Frequency of processed food consumption (secondary outcome)**:<br>o The **unadjusted estimates** for the relationship between the **frequency of processed food consumption** and **periodontal health** revealed significant associations for several food categories:<br>▪ **Packaged milkshakes**: p = 0.003;<br>▪ **Industrial juices**: p = 0.009;<br>▪ **Industrial pastries**: p = 0.018;<br>▪ **Fruits in syrup**: p = 0.022.<br><br>These unadjusted results show that **higher consumption of processed foods** was associated with **worse periodontal health**. The estimates of association for processed foods were	16	(*a*) Give unadjusted estimates and, if applicable, confounder-adjusted estimates and their precision (eg, 95% confidence interval). Make clear which confounders were adjusted for and why they were included<br>(*b*) Report category boundaries when continuous variables were categorized<br>(*c*) If relevant, consider translating estimates of relative risk into absolute risk for a meaningful time period

more pronounced in boys, where the following food categories showed significant associations with periodontal health:

- **Packaged milkshakes**: p = 0.044;
- **Salty snacks**: p = 0.032;
- **Cold cuts**: p = 0.033.

2. Confounder-adjusted estimates:

- To assess whether the observed associations between processed food consumption and periodontal health remained after adjusting for potential **confounders**, the following variables were included in the regression model:
  - **Sex**: given the significant difference observed between boys and girls in terms of periodontal health, sex was adjusted for to account for sex-based variations in periodontal disease and food consumption.
  - **School type**: since participants were drawn from both public and private schools, **school type** was adjusted for to ensure that differences in socioeconomic factors or access to certain foods did not influence the results.
  - **Geographic location**: geographic location (urban vs. rural) was considered to capture differences in food access, diet, and lifestyle that could influence periodontal health.
  - **Socioeconomic status (if available)**: this could influence both food consumption patterns and access to dental care.

Other analyses:<br><br>No other analyses were carried out.	17	Report other analyses done—eg analyses of subgroups and interactions, and sensitivity analyses
**Discussion**		
Key results:<br><br>The findings of this study emphasize the important role of **dietary habits** in the development of **periodontal disease** among adolescents, particularly with regard to the consumption of **ultra-processed foods** and **sugary products**. This study highlighted several key outcomes aligned with the study objectives and confirmed known associations between diet and oral health, as well as observed sex-based differences in food consumption and periodontal health.	18	Summarise key results with reference to study objectives
Limitations:<br><br>While the findings are insightful, this study has some limitations. The sample size of **233 adolescents** may not be fully representative of the	19	Discuss limitations of the study, taking into account sources of

broader population, limiting the generalizability of the findings. Moreover, being a **cross-sectional study**, it cannot establish **causal relationships** between food consumption and periodontal disease. The reliance on **self-reported data** may introduce potential **recall bias**, and **confounding factors** such as oral hygiene habits and **socioeconomic status** were not fully controlled, which may have influenced the results.		potential bias or imprecision. Discuss both direction and magnitude of any potential bias
Interpretation:<br><br>The results of this study offer valuable insights into the relationship between **dietary habits**, particularly the consumption of **ultra-processed foods**, and the development of **periodontal disease** among adolescents. However, as with any observational study, caution must be exercised when interpreting these findings, considering this **study's limitations**, the **multiplicity of analyses**, and the context provided by similar studies and existing evidence.	20	Give a cautious overall interpretation of results considering objectives, limitations, multiplicity of analyses, results from similar studies, and other relevant evidence
Generalizability:<br><br>While this study provides important findings about the relationship between ultra-processed food consumption and periodontal disease in adolescents, its generalizability is somewhat limited. The results are particularly relevant to populations with dietary habits like those of the study sample, such as those in Spain or regions with high consumption of processed foods. However, the findings may not directly apply to adolescents in other regions with different dietary patterns, levels of socioeconomic inequality, or access to healthcare and oral health education.	21	Discuss the generalisability (external validity) of the study results
**Other information**		
Funding:<br><br>This work was supported by the ADEMA+ Foundation. The original study on which the present article is based is as follows: Coll, I.; Vallejos, D.; Cuesta, R.; Domínguez, J.; Tomás, P.; López-Safont, N. Prevalence of Oral Diseases and the Influence of the Presence of Overweight/Obesity in Schoolchildren Population in Mallorca. J. Clin. Med. 2024, 13, 7283. https://doi.org/10.3390/jcm13237283	22	Give the source of funding and the role of the funders for the present study and, if applicable, for the original study on which the present article is based

Note: An Explanation and Elaboration article discusses each checklist item and gives methodological background and published examples of transparent reporting. The STROBE checklist is best used in conjunction with this article (freely available on the Web sites of PLoS Medicine at http://www.plosmedicine.org/, Annals of Internal Medicine at http://www.annals.org/, and Epidemiology at http://www.epidem.com/). Information on the STROBE Initiative is available at www.strobe-statement.org.
